# Microbiome and cardiovascular health unexplored frontiers in precision cardiology: a narrative review

**DOI:** 10.1097/MS9.0000000000003430

**Published:** 2025-05-26

**Authors:** Anano Nebieridze, Aya Abu-Bakr, Abubakar Nazir, Abir Ghosson, Anamarija Minova, Olivier Uwishema

**Affiliations:** a Department of Research and Education, Oli Health Magazine Organization, Kigali, Rwanda; b Faculty of Medicine, David Tvildiani Medical University, Tbilisi, Georgia; c Biotechnology Department, Faculty of Agriculture Science, Ain Shams University, Shubra Al Kheimah, Awal Shubra Al Kheimah, Al Qalyubia Governorate, Cairo, Egypt; d Faculty of Medicine, Beirut Arab University, Beirut, Lebanon; e Faculty of Medical Sciences, University of “Goce Delchev”, Shtip, Republic of North Macedonia

**Keywords:** cardiovascular disease, cardiovascular health, gut-heart axis, precision cardiology

## Abstract

**Background and purpose:**

Gut microbiota has a symbiotic relationship with their host. It is known that the gut microbiome has the potential to affect the host and vice versa. Cardiovascular disease and its comorbidities are the leading cause of death worldwide. Patients with various heart conditions have been observed to have a different composition of the gut microbiome. It has been postulated that the gut microbiome and its derivatives exert various effects on the cardiovascular system, termed the gut-heart axis. In this study, we aim to explore how the gut microbiome and the active metabolites produced by these microorganisms affect patient cardiovascular health. Additionally, we will discuss how gut microbiota can become a target for the new era of precision cardiology.

**Methods:**

Data were collected through the online databases PubMed, Google Scholar, Ovid MEDLINE, and ScienceDirect. Articles regarding cardiovascular health and pathology as well as its overlap with gut microbiome and health were used.

**Results:**

Emerging evidence suggests that gut microbiome has a significant influence on cardiovascular disease through its metabolites, such as trimethylamine N-oxide and short-chain fatty acids, which impact cholesterol metabolism, systemic inflammation, and plaque stability. Targeting said derivatives has proven to provide beneficial results for patients suffering from cardiovascular disease.

**Conclusions:**

Finding reported here highlights the importance of microbiome in cardiovascular disease and health and suggest that microbiome-based interventions hold promise for prevention and treatment of cardiovascular disease. More research needs to be conducted to study more concrete effects of specific microorganisms on cardiovascular health. Multicenter, longitudinal studies with a large sample size will provide the best evidence for clinically significant findings. Using precision cardiology, to target the gut microbiome and its derivatives, with medications like antibiotics, and nonpharmacologic interventions like lifestyle modification and fecal transplantation can positively influence cardiovascular health and help with the effective management of ongoing diseases.

## Introduction

Human microbiota studies have expanded our understanding of the paramount importance of microbiota for health across all stages of life. A symbiotic relationship exists between the human microbiota and the host at the locations where microbial colonization has occurred, such as the oral cavity, intestinal tract, skin, and genitourinary tract. This relationship helps in sustaining the physiological homeostasis of the host^[1]^.

**HIGHLIGHTS**

- The gut microbiota (GM) plays a part in the pathogenesis of many diverse diseases, including cardiovascular diseases (CVDs). CVD, with atherosclerosis being a primary manifestation, is one of the leading causes of mortality worldwide, with over 17.9 million deaths in 2019.
- The GM can influence both the regulation of cholesterol metabolism in the liver and systemic cholesterol levels, by playing a role in altering bile acids.
- Research and prevention/treatment options are needed because the association between the cardiovascular system and overall health is getting more and more complicated. Personalizing/customizing based on people’s experience could lead to new preventive strategies and new treatments that would reduce the chance of CVD.

The gut microbiota (GM) plays a role in the pathogenesis of many diverse diseases, including cardiovascular diseases (CVDs)^[2]^. CVD, with atherosclerosis being a primary manifestation, is one of the leading causes of mortality worldwide, with over 17.9 million deaths in 2019^[3,4]^. New data exploring factors implicated in the development of atherosclerosis suggest GM to be a primary player, due to the contribution the microbes’ metabolites (i.e. bile acids, trimethylamine N-oxide [TMAO], and short-chain fatty acids [SCFAs]) have in cholesterol homeostasis^[5]^. The GM can influence both the regulation of cholesterol metabolism in the liver and systemic cholesterol levels by playing a role in altering bile acids. Coronary artery disease (CAD) is the most prevalent form of CVD, and high serum cholesterol (hypercholesterolemia) is a well-documented risk factor associated with the development of CAD^[6]^.

The primary goal of this review is to explore the emerging evidence on the gut-heart axis and its unique role in the pathogenesis of CVD. Specifically, this review will focus on highlighting mechanisms through which GM influences cardiovascular health of the host, discussing the potential adverse health effect exerted by changes in the gut microbiome, evaluating challenges and barriers encountered in studying the complex interplay between the microbiome and CVD, while exploring potential strategies to overcome these challenges, and emphasizing the importance of further exploration of microbiota-targeted interventions as a novel approach for precision cardiology.

### Human microbiota

The human microbiota is defined as all organisms that normally live on the inner and outer surfaces of a human body and it consists of bacteria, archaea, viruses, and eukaryotes. These microorganisms’ effect on human physiology, both in health and in disease, help in strengthening or weakening metabolic and immune functions^[7]^. The symbiotic interaction between the human body and its natural microbiota begins at birth and it plays a critical role in sustaining overall human health and well-being^[7]^.

The intestinal mucosa is considered one of the largest immunologically functional organs in the human body; it defends its host from invasive microorganisms. The majority of bacterial species that are located in adult human and mouse guts belong to the phyla *Firmicutes* and *Bacteroidetes*, rarer organisms include bacterial phyla such as *Actinobacteria, Fusobacteria, Verrucomicrobia*, and *Proteobacteria*^[8]^. Thankfully, while most of these microorganisms support host health, disruption in microbial balance – known as dysbiosis – can drive several gastrointestinal and extra-intestinal disorders^[8,9]^. Dysbiosis occurs when stressful conditions disrupt the natural microbial balance, acutely decrease microbial diversity, and promote the expansion of a specific bacterial type. A combination of natural variations and stress factors mediate these disruptive events, thus the exact mechanism remains vague^[10,11]^. Oxidative stress, bacteriophage induction, and the secretion of bacterial toxins can trigger rapid shifts among intestinal microbial groups, resulting in dysbiosis^[11]^. Other factors influencing the homeostasis of the microbiota are diet, age feeding pattern, and antibiotics^[9,11]^.

Several factors can influence the composition of the gut microbiome, including, host’s age, diet, feeding pattern, and antibiotic use. Host-related factors also play a key role, as various molecular signals produced by the host can alter the composition of its cellular surface structures^[9]^. Another regulatory element is microRNAs (miRNA). miRNAs are small (about 18–23 nucleotides in length) non-coding RNAs. Fecal miRNAs, in particular, can be used to influence the GM. A procedure called fecal transplantation serves the purpose of introducing donor fecal material into the recipient. After fecal miRNA is transplanted, the recipient’s microbiota profile resembles that of the donor, as demonstrated in the transfer of healthy mouse donors to IEC-deficient mice studies^[10]^. This finding reveals a promising avenue for future treatments, where synthetic specific miRNA could be used to address diseases linked to GM alterations^[9]^.

### Cardiovascular health and disease

CVD is a general term used to describe various conditions that affect the cardiovascular system^[12]^. In the United States and industrialized societies, CVD is the leading cause of death^[13]^. CVD is responsible for roughly one in every three deaths in the U.S. and one in every four deaths in Europe^[14]^. Rupture of the atherosclerotic plaques, located in the intima of many middle or large arteries can cause catastrophic consequences, such as myocardial infarction (MI), heart failure (HF), claudications, and stroke. Various factors influence the risk of CVD, including genetic and environmental sources, either separately or in combination^[4]^. There is a steady increase in certain risk factors for CVDs, for example, type 2 diabetes mellitus, metabolic syndrome, and obesity^[15]^. Less than one-fifth of the cardiovascular risk is attributable to genetic parameters, despite extensive research aimed at identifying causal genetic variants through large-scale genome-wide association studies^[13]^.

A study by Yusuf *et al*^[15]^ indicates that there is a potential modifiable risk factor that accounts for the majority of CVD. Although some risk factors have a significant global impact (e.g. hypertension, low education, tobacco), the impact of others (e.g. household air pollution, poor diet) varies by the socioeconomic status of countries. This highlights the importance of adjusting various health recommendations and policies to suit individual counties’ needs and unique risk factor exposure^[15]^.

For the primary prevention of CVD, the individual should adopt the general approaches issued by their country of origin and follow local guidelines published by local professional medical authorities. A healthy lifestyle, regular check-ups, and proper therapy for conditions associated with elevated risk of CVD, such as diabetes, hypertension, and hyperlipidemia, should be maintained^[16]^. The current, new, and promising approach for the treatment of CVDs is targeted therapy. Researchers can analyze the pathogenesis of these diseases and investigate targeted therapy by using proteomics, genomics, and transcriptomics, to bring the treatment of CVDs into a precision era. There is a huge necessity for further investigation to overcome the side effects and the unexpected off-target event issue^[17]^.

### Emerging evidence on the microbiome-cardiovascular health axis

#### Atherosclerosis and CAD

Existing literature highlights the influence of GM composition on atherosclerosis and CAD^[18]^. Intestinal barrier disruption due to alteration in GM – one of the mechanisms behind this association – is mainly caused by ammonia and ammonium hydroxide, formed through the hydrolyzation of urea by gut microorganisms^[19]^. This disruption leads to migration of gut microorganisms in the atherosclerotic plaques. Presence of bacterial DNA in atherosclerotic plaques is supported by various studies. Ziganshina *et al*^[20]^ have identified bacterial DNA in all studied atherosclerotic plaques, with notable presence of *Burkholderiales*, including Curvibacter as well as other genera. Similarly, Lindskog Jonsson *et al*^[21]^ confirmed the presence of bacterial DNA in atherosclerotic plaques using quantitative PCR analysis. Presence of bacterial DNA in the atherosclerotic plaques has been associated with potential inflammatory process and worsening the patient’s condition^[22–24]^. While studies, such as those mentioned above, identified presence of bacterial DNA in atherosclerotic plaques, it’s effect on plaque stability and inflammation remains unclear. Ziganshina *et al*^[20]^ identified bacterial communities, including members of the order Burkholderiales, which may influence the local inflammatory environment within plaques. Koren *et al*^[22]^ found specific bacterial taxa, such as *Chryseomonas* and *Veillonella*, in plaques, suggesting that these bacteria might contribute to inflammatory processes that affect plaque stability. Szulc *et al* identified periodontopathic bacteria like *Porphyromonas gingivalis* in plaques, which are known to induce inflammatory responses and could potentially exacerbate plaque vulnerability^[22]^. Increased presence of pro-inflammatory peptidoglycans and decreased levels of anti-inflammatory carotene also contributed to plaque instability^[25]^. Additionally, it is reported that levels of *Roseburia* bacteria in fecal materials are minimized among patients with unstable atherosclerotic plaque compared to those with stable ones^[25]^. Use of probiotics has a potential to improve outcomes in patients with atherosclerotic plaques. Several studies have demonstrated that probiotic use can positively influence atherosclerosis by modulating inflammation, oxidative stress, and lipid metabolism. For example, Lab4P consortium of probiotics has been shown to decrease plaque burden and lead to plaque stabilization^[26–28]^. The interconnection between microbiota composition and atherosclerosis not only influences plaque stability but also impacts post-MI complications. Studies showed that using probiotics decreases the risk of a patient developing HF post-MI^[29]^.

#### Heart failure

Concerning HF, many articles illustrated the interconnection between gut dysbiosis and the development of HF^[30,31]^. Literature emphasizes that gut barrier instability and leakage of various microorganisms and their toxic products are considered to be two of the main mechanisms behind this association^[30]^. Studies have shown that HF patients exhibit a distinct GM profile compared to healthy individuals. Notably, these patients have a decrease in beneficial bacteria (e.g. *Faecalibacterium prausnitzii*) and an increase in potential pathogenic bacteria such as *Ruminococcus gnavus*^[32]^. The imbalance leads to increased intestinal permeability, allowing certain bacterial translocation and passage of bacteria-derived metabolites into the bloodstream. These metabolites can potentially contribute to systemic inflammation and exacerbate HF^[33,34]^. Microbiota migration accelerates the chronic inflammatory state among HF patients, thereby altering the normal function of heart muscles^[30]^. Moreover, gut-derived metabolites such as TMAO have been implicated in HF pathology. Elevated levels of TMAO, produced by gut bacteria, are associated with adverse cardiovascular outcomes^[34]^. On the other hand, in individuals, particularly those with a decompensated HF, the disturbance of the typical composition of gut microbial communities occurs due to intestinal hypoperfusion, resulting in alterations in local pH and luminal hypoxia within the gut^[35]^.

#### Hypertension

Regarding hypertension, multiple studies demonstrated the link between changes in the normal flora of the gut and blood pressure control^[36,37]^. Studies illuminated that the microbial plenitude and diversity decreased significantly in hypertensive patients compared to normal individuals, while certain harmful bacteria such as *Prevotella* and *Klebsiella* were found to be more abundant^[36]^. Moreover, the relation between hypertension induced by high salt intake and Lactobacillus was explained in literature; *Lactobacillus* levels are reduced among people with high salt diet intake, and this bacterium helps regulate blood pressure among salt-sensitive hypertensive patients^[37]^. Hypertensive patients exhibit reduced microbial diversity and an increased Firmicutes/Bacteroidetes ratio, which is indicative of dysbiosis. This imbalance is associated with decreased levels of beneficial bacteria that produce SCFAs like acetate and butyrate, which have anti-inflammatory properties^[38–40]^. Similarly to HF, hypertension can lead to increased gut permeability, allowing for translocation of bacterial products and trigger systemic inflammation. This process can contribute to elevated blood pressure^[39–42]^.

#### TMAO and SCFAs

Studies showed that TMAO and SCFAs are two by-products produced by GM and are implicated in CVD development through distinct mechanisms. TMAO, a pro-inflammatory microbial metabolite, formed by the oxidation of trimethylamine (TMA) in the liver by the action of an enzyme called hepatic flavin, has emerged as a potent biomarker and contributor to atherosclerosis. TMA is formed through the GM-mediated metabolism of dietary phosphatidylcholine and carnitine^[43]^. TMAO promotes cholesterol accumulation in macrophages enhanced by the formation of foam cells^[44,45]^. Additionally, it impairs reverse cholesterol transport and decreases biliary cholesterol excretion, collectively fostering plaque formation and progression. Elevated levels of TMAO have been associated with increased risk of atherosclerosis and adverse cardiovascular events due to macrophage activation, endothelial dysfunction, and vascular inflammation^[46–49]^. Furthermore, TMAO exacerbates vascular inflammation and endothelial dysfunction, precipitating the development of cardiovascular complications^[44,50]^.

Conversely, SCFAs – primarily acetate, propionate, and butyrate – are microbial dietary fiber fermentation end-products in the colon^[51]^. SCFAs exert protective effects against CVD development by modulating various metabolic and inflammatory pathways^[52]^. Butyrate, for instance, acts as a histone deacetylase inhibitor, regulating gene expression involved in lipid metabolism and inflammation^[53]^. Moreover, SCFAs affect insulin sensitivity and metabolic homeostasis by acting on both colonocytes and hepatocytes^[54]^. Through their multifaceted actions, SCFAs mitigate atherosclerosis progression, reduce systemic inflammation, and improve endothelial function, all of which translate to improved cardiovascular health^[55]^. Dysbiosis can lead to altered SCFA production and contribute to increased intestinal permeability and systemic inflammation, which are risk factors for CVD^[56,57]^.

Understanding the intricate effects of TMAO, SCFAs, and other by-products of gut microbiome activity, allowing us to develop new therapeutic modalities for the management of CVD.

#### Methodological challenges and future directions

In evaluating the current studies, it’s important to highlight the limitations physicians face and the potential avenues for improvement. Various studies aiming to show the association between the gut’s normal inhabitants and cardiovascular health have numerous limitations, related to study types, methodologies, and the complexity of the GM. The majority of the studies conducted used a small sample size and had a single-center focus, which limited the diversity of the participants’ characteristics. This design restricted the findings and limited their generalizability. People’s normal gut flora differs depending on the age, diet, physical activity, patient’s health and comorbidities, and the use of prescribed or over-the-counter medications. Targeting a small sample makes it more challenging to match the participants by the possible confounders and decreases the power of the study. Hence, going forward, it is vital to work with large samples consisting of diverse traits, allowing generalizability and a deep comprehension of findings related to dysbiosis and CVDs.

Moreover, another limitation encountered while assessing the interlink between microbiota and CVDs was the presence of many studies conducted on animal species. While animals play an important role in research and can be a part of understanding various mechanisms of different medical topics, challenges arise when trying to translate certain findings in animal models to human subjects^[58,59]^. The translational gap between both studies depends on different circumstances such as study design and methodology. Studies conducted on animals are usually tightly controlled and conducted in strictly regulated environments. In contrast, similar studies conducted on humans are not as uniform and have a lot of outside factors that could disturb the homogenous system, required for the accurate assessment of the results. Demographics, dietary habits, physical activity, and subjects’ health and well-being are just some of the factors that are encompassed while conducting human trials. Therefore, directing our studies more toward humans would be of great value in establishing the association between cardiovascular health and gut flora.

Furthermore, establishing a cause-and-effect relationship between the gut microbiome and CVD has proven to be extremely difficult. Determining causality required longitudinal studies conducted over extended periods of time to track changes in the composition of gut microbiome. Moreover, detecting a positive or negative impact of a specific gut microorganism on cardiovascular health requires highly specialized tools capable of detecting which microorganisms determined a specific outcome. Additionally, randomized controlled trials are required to assess the impact of various interventions such as prebiotics, probiotics, and antibiotic intake, and fecal microbiota transplantation (FMT) on cardiovascular health.

Finally, it is noteworthy to mention that all of what has been discussed before could be related to the fact that GM is a complex ecosystem that is diverse and individualized. Its composition varies between different geographical areas, ethnicities, and health-related practices^[60]^. Therefore, by including a wide range of participants and integrating data from diverse populations, researchers can conclude and popularize findings related to the microbiota cardiovascular health axis.

### Implications for precision cardiology

#### Opportunities for precision medicine in cardiovascular health

Precision medicine is transforming cardiovascular health by tailoring treatments to an individual’s genetics, environment, and lifestyle. This approach enhances efficacy, reduces risks, and improves outcomes^[61,62]^.

### Potential role of microbiome-targeted interventions in CVD prevention and treatment

One of the key advantages of precision medicine in cardiovascular care is its ability to enable accurate risk assessment and early detection of cardiovascular conditions. By identifying detailed genetic information and other personalized factors, clinicians can identify individuals at heightened risk of developing CVDs. Its vital role in identifying genetic predispositions to CVDs provides valuable insights into an individual’s inherent risk factors. Armed with this knowledge, healthcare providers can implement targeted strategies, such as lifestyle modifications and pharmacogenomic therapies, to mitigate disease progression and optimize patient outcomes. By tailoring interventions to address specific genetic vulnerabilities, precision medicine holds immense promise in ushering in a new era of personalized cardiovascular care. This approach allows for timely interventions and preventive measures, ultimately reducing the burden of CVD on individuals and healthcare systems^[62]^.

Several interventions can provide improved cardiovascular effects by modulating the gut microbiome. These interventions can be further subdivided into either lifestyle modifications or targeted interventions.

Dietary modification seems like a minor intervention but, in reality, has a significant impact on cardiovascular health, through its effects on the gut microbiome. A balanced diet, that is rich in various nutrients and also contains an ample amount of fruits, vegetables, whole grains, soluble fibers, lean proteins, and healthy fats, positively affects the composition of the gut microbiome^[63]^. Also referred to as the Mediterranean diet, this meal compositing has proven to significantly lower the risk of developing CVD. Fiber-rich foods also offer another proven benefit; by promoting the proliferation of acetate-producing organisms and suppressing opportunistic microbe propagation, they help reduce blood pressure and mitigate pathologic remodeling of the heart associated with hypertension^[64]^. Additionally, the Mediterranean diet provides various nutrients to keep the beneficial gut bacteria thriving, which conversely produce SCFAs that exert anti-inflammatory effects and help maintain adequate cardiovascular health^[65]^.

The use of probiotics, prebiotics, and synbiotics also seem to confer various beneficial effects on the cardiovascular system, ranging from decreased cholesterol levels to reduced systolic and diastolic blood pressures^[65–68]^. Probiotics are live microorganisms that can be consumed by the host. These microorganisms, in turn, exert beneficial effects onto the host organism. The most common probiotics are Lactobacillus, Streptococcus, and Enterococcus species^[69]^. Several studies have shown that consistent use of probiotics has a positive effect on reducing systolic and diastolic blood pressure. Similarly, prebiotics, which are non-digestible fibers (e.g. inulin), can promote the growth of beneficial gut bacteria. Finally, synbiotics, which are a combination of pro- and prebiotics, provide the combined beneficial effect mentioned above.

Antibiotics, though having the potential to upset the microflora of the intestines, in certain situations and with targeted therapy, may be helpful to lessen inflammation brought on by a particular intestinal bacterium. By targeting harmful bacteria, it is possible to reduce inflammatory markers that propagate CVD^[65–68]^. To reduce the risk of antibiotic resistance developing as well as collateral harm to helpful bacteria, antibiotic selection must be carefully considered. Additionally, taking probiotics with antibiotics may help protect the microflora, preventing it from doing as much harm.

FMT was first developed to treat Clostridium difficile infection. The procedure involved transferring fecal material from a healthy donor to the intestinal tract of the recipient. This is done in an attempt to restore the balance of the beneficial gut microflora. FMT can be performed through various means, including oral capsules or colonoscopy enema. The most fit and appropriate donor must be selected to ensure the positive outcome of the procedure.

Good digestion is linked to good heart health^[63]^. Microbiome modification holds the potential for enhancing patient outcomes and lessening the burden of CVDs when incorporated into individualized treatment regimens. Furthermore, more study is required to maximize the effectiveness and safety of microbiome-based therapies as well as to comprehend the mechanisms by which the GM affects cardiovascular health. Additionally, educating patients about their particular cardiovascular risks and available personalized treatments can increase patient involvement and treatment plan adherence. Patients become active participants in their healthcare journey, which improves overall well-being, leads to better outcomes, and fosters shared and collaborative decision-making processes.

### Future directions for research and clinical practice

In addition to genetic factors, precision medicine also sees the importance of environmental and lifestyle influences on cardiovascular health. By implementing these considerations into treatment plans, clinicians can adopt a holistic approach that addresses the root causes of CVDs. Mitigating the adverse effects of various CVD medications and improving patient outcomes by targeting specific factors responsible for the disease development can greatly improve patients’ quality of life^[70,71]^.

## Summary

This review explores the gut-heart axis and its role in CVD development and progression. Microbiome research is paramount for understanding how the human body functions, assessing disease risks, and predicting potential health outcomes. While particular effects are not yet clear, certain alterations in gut microbiome have been positively correlated with CVD through expression of bacterial metabolites like TMAO and SCFAs; a disbalance between which contributes to systemic inflammation and plaque instability. However, much remains unknown. The review addresses the limitations of current studies and suggests strategies to overcome them, including larger human studies and inclusion of microbiome-targeted therapies. It emphasizes the need for continued research into microbiome-based interventions, which could be valuable for precision cardiology by enabling personalized interventions and improving patient outcomes.

## Data Availability

Not applicable.
